# Influence of age on results following surgery for displaced acetabular fractures in the elderly

**DOI:** 10.1186/s12891-017-1817-5

**Published:** 2017-11-25

**Authors:** Guo-Chun Zha, Xue-Mei Yang, Shuo Feng, Xiang-Yang Chen, Kai-Jin Guo, Jun-Ying Sun

**Affiliations:** 1 Department of Orthopedic Surgery, the Affiliated Hospital of Xuzhou Medical University, No. 99 Huaihai West Road, Xuzhou, Jiangsu 221002 People’s Republic of China; 2 Hyperbaric Oxygen Treatment Center, the Affiliated Hospital of Xuzhou Medical University, No. 99 Huaihai West Road, Xuzhou, Jiangsu 221002 People’s Republic of China; 3 Orthopaedic Department, the First Affiliated Hospital of Soochow University, 188 Shizi Street, Suzhou, Jiangsu 215006 People’s Republic of China

**Keywords:** Acetabular fracture, Elderly patients, Age, Outcomes

## Abstract

**Background:**

Elderly patients have more special medical needs when compared with young ones; thus, the results of open reduction and internal fixation (ORIF) for acetabular fractures should be stratified by age in these patients. This study seeks to determine whether the age of the patient influences the results of the ORIF for acetabular fractures.

**Methods:**

We performed a retrospective analysis of prospectively collected data on 53 elderly patients with displaced acetabular fractures who underwent ORIF between May 2004 and May 2011. Patients were divided into two groups by age: young–old group (60–74 years) and old–old group (75–90 years). The number of patients in each group was 28 and 25. The reduction quality and clinical function was evaluated using the Matta criteria and modified Postel Merle D’Aubigne Score, respectively. Operative time, bleeding amount, and complications were recorded.

**Results:**

Patients in old–old group had significantly lower anatomical reduction rate (*p* = 0.024), less operative time (*p* = 0.021), and less bleeding amount (*p* = 0.016) than those in the young–old group. The reduction quality in the young–old group was strongly associated with clinical function (*p* < 0.05). However, no difference in clinical function was detected among the different reduction qualities in the old–old group (*p* > 0.05). Moreover, no significant difference in clinical functions (*p* = 0.787) and complications (*p* = 0.728) was detected between the two groups.

**Conclusions:**

Old–old patients may expect comparable clinical functions and complications with young–old patients. The reduction quality in old–old patients may be not significantly associated with clinical function. Different treatment strategies may be applied for acetabular fractures with ORIF in different age groups.

**Electronic supplementary material:**

The online version of this article (10.1186/s12891-017-1817-5) contains supplementary material, which is available to authorized users.

## Background

The incidence of acetabular fractures in the elderly has increased [1]. However, the optimal management of displaced acetabular fractures in elderly patients remains controversial. Some surgeons prefer advocated conservative treatment for elderly patients with limited physiological reserves and medical comorbidity that may increase the risk of surgery [2, 3]. Other surgeons advise total hip arthroplasty (THA) for elderly patients with poor bone quality that may increase the risk of poor reduction and loss of reduction or failure of fixation or both [4–6]. Considering the limited life expectancy, low activity level, and low functional requirements of elderly patients, some authors believe that open reduction and internal fixation (ORIF) is suitable for these patients [1, 7, 8].

The inconsistent clinical results can be attributed in part to the following reasons: (1) previous studies included patients of a wide age variety, i.e., greater than 40 [9], 50 [10], 55 [11] to 60 [1] and 70 years old [8]; and (2) the results of studies that classified elderly patients as a single population were not stratified by age. Elderly patients have more special medical needs when compared with young ones; thus, different age groups of elderly patients may present different physiological reserves, lifestyle qualities, and osteoporosis degrees. Accordingly, the results of acetabular fractures in the elderly should be stratified by age. Extracting practical conclusions about the relationship of age, reduction quality, and clinical function after ORIF is difficult without a comparator group.

To the best of our knowledge, none of studies have focused exclusively on the relationship of the age to clinical function, reduction quality, and complication of ORIF for acetabular fracture in elderly patients. Therefore, this study seeks to determine whether the age of the patient influences the results of the ORIF for acetabular fractures.

## Methods

### Patient source

We performed a retrospective analysis of prospectively collected data on 66 consecutive elderly patients with displaced acetabular fractures between May 2004 and May 2011. Four patients were conservative therapy, and 62 patients underwent ORIF. The 62 patients were divided into two groups by age: young–old group (60–74 years) and old–old group (75–90 years). This study received approval from the institutional review board. Informed consent was obtained from all patients included in the study.

### Surgical technique

All surgeries were performed by the senior author (J.Y.S) at an average of 5.8 ± 2.5 days (range, 2–14 days) from the initial injury. Skeletal traction was selectively employed through the distal femoral pin. The medical conditions of the patients were optimized through a preoperative multidisciplinary approach. Despite the absence of specific intraoperative management criteria, the surgeon closely followed the principles that accurate reduction is attempted for young–old patients with an active and good physical state. Limited operative procedure was used for old–old patients with low activity and functional requirements to achieve the stable internal fixation of fractures and prevent anatomical reduction.

All patients were placed in floppy lateral position and then operated under general anesthesia. The approach (Kocher–Langenbeck, Ilioinguinal, or Kocher–Langenbeck + Ilioinguinal) was decided according to the nature of the acetabular fracture and the preference of orthopedic surgeon (J.Y.S).

### Perioperative regimen

All patients received adequate pain control during the perioperative period. The drain was removed after 48 h, and three standard plain radiographs were obtained after 3 days. On the day after surgery, the patients were encouraged to sit up in bed and perform passive functional exercises to strengthen the hip joint, quadriceps, and hamstrings. The patients were allowed touch-toe weight-bearing a maximum of 20 kg for the first 12 weeks with a walker or crutches depending on physical state. After 12 weeks, full weight-bearing gradually progressed according to patient tolerance.

### Follow-up

After discharge, the patients were traced using telephone, letter, or e-mail and were asked to return for completing the clinical and radiological postoperative evaluation at 6 weeks, 3 months, 1 year, and annually thereafter. Patients who refused or were unable to return were contacted by two surgeons (S.J.D and J.X.T) to obtain the information in their home.

### Data collection

The information included comorbidity, mechanism of injury, fracture type, associated injury, operative time, bleeding amount (including operative blood loss and wound drainage), operative approach, complications, quality of reduction, clinical function, and radiological outcomes. The results from patients who were confirmed to have died from natural causes after 12 months follow-up was included in this study. By contrast, the results from patients who had been lost to follow-up or confirmed to have died from natural causes within 12 months follow-up were excluded. Data from the final follow-up visit were analyzed for this study.

### Radiographic and clinical evaluation

Three standard plain preoperative radiographs (anteroposterior pelvis, obturator oblique, and iliac oblique views) were obtained along with computed tomography scans to classify fracture according to Letournel’s classification system [12]. Reduction quality was evaluated on the three-standard plain post-operative radiographs and graded as anatomical (0 mm to 1 mm of displacement), imperfect (2 mm to 3 mm displacement), or poor (more than 3 mm displacement) on the basis of the residual displacement as defined by Matta [9].

Clinical function was categorized as excellent (18 points), good (15–17 points), fair (13–14 points), or poor (< 13 points) on the basis of the modified Postel Merle D’Aubigne Score, which includes degree of pain (0–6 points), degree of ambulation (0–6 points), and range of motion (0–6 points) components [9, 13]. Radiological outcome was evaluated on the follow-up postoperative radiographs and was graded as excellent, good, fair, and poor in accordance with Matta criteria [9].

The acetabular fracture types were classified by a senior author (J.Y.S). Reduction quality was evaluated by two surgeons (S.K.Z and Z.S.Z) who did not participate in any of the surgical procedures. Two other surgeons (S.J.D and J.X.T) who were blinded to reduction quality completed the clinical and radiological evaluation. The senior author (J.Y.S) made the final assessment when the results (reduction quality and radiological outcome) were inconsistent. We take the average of the clinical outcomes that were evaluated by the two surgeons.

### Statistical analysis

Analysis was performed using STATA version 11.0 (StataCorp LP, College Station TX). Continuous data were expressed as mean ± standard deviation (SD) and were analyzed using t-test or one-way ANOVA. Tukey’s test was performed for multiple comparisons. The ordinal data were analyzed with Kruscal–Wallis test. Statistical significance was considered at *p* < 0.05 unless otherwise specified.

## Results

### Patient characteristics

Of the 62 patients, 7 (11.3%) were lost to follow-up at a mean of 18 months (6–31 months) postoperatively and 2 (3.2%) had died from natural causes within 12 months follow-up, during which all patients had excellent or good clinical function. The nine patients were excluded from this study. The remaining 53 patients (44 males and 9 females) aged 60–90 (mean age: 72.8 ± 6.6 years) were enrolled. The mean follow-up was 52.5 ± 24.1 months (range: 18–110 months). Detailed distribution of patient demographics and characteristics, fracture types, mechanism of injury, and associated injury is shown in Table 1.

**Table 1:** Demographics of both groups

Variable	Young-old group (*n* = 28)	Old-old group (*n* = 25)
Mean age (years)	67.8 ± 4.1 (60–74)	78.5 ± 3.5 (75–90)
Sex ratio (male:female)	25: 3	19: 6
Mean BMI (kg/m^2^)	22.3 ± 2.8 (17.9–25.5)	22.3 ± 2.1 (21.3–24.8)
Comorbidity (n)		
Hypertension	15	18
Anemia	6	9
Cardiopathy	2	4
Diabetes mellitus	3	2
Pulmonary	1	4
Cerebrovascular accident	1	3
Fracture types		
Associated both column	9	9
T-shaped	6	3
Transverse + posterior wall	5	2
Anterior column	1	4
Posterior wall	3	2
Anterior wall	0	4
Transverse	2	1
Posterior column	1	0
Posterior column + posterior wall	1	0
Mechanism of injury		
Fall at home	2	7
Pedestrian	1	10
Bike accident	2	1
Motorcyclist	4	0
Auto vs. Pedestrian	14	5
Fall from >2 m	5	2
Associated injury		
Splenic rupture	1	0
Sacroiliac joint dislocation	1	0
Thoracic spine and costal fracture	1	0
Head trauma	1	0
Mean follow-up (months)	60.7 ± 26.6 (24–110)	43.3 ± 17.2 (18–84)

### Operative variable

Operative approach, operative time, and bleeding amount are shown in Table 2. Anatomical reduction was observed in 28 patients (52.8%), imperfect in 19 (35.8%), and poor in 6 (11.3%). Compared with the old–old patients, the young–old patients were more likely to achieve anatomical reduction (67.9% vs. 36%, *p* = 0.024) (Table 2) but more blood loss (705 mL vs. 591 mL, *p* = 0.016) and longer operative time (145 min vs. 110 min, *p* = 0.021) (Table 2).

**Table 2:** Operative parameters of both groups (*n* = 53)

Variable	Young-old group (n = 28)	Old-old group (n = 25)	*p*-value
Operative approach			0.022
Kocher-Langenbeck	7	4	
Ilioinguinal	1	8	
Kocher-Langenbeck + Ilioinguinal	20	13	
Mean operative time (min)	145 ± 53	110 ± 54	0.021
Mean bleeding amount (ml)	705 ± 308	591 ± 204	0.012
Quality of reduction			0.024
Anatomic	19	9	
Imperfect	7	12	
Poor	2	4	

### Clinical results at the final follow-up

**Fig. 1:**
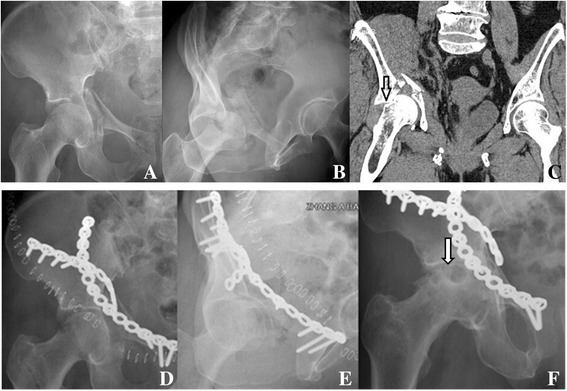
Radiographs of a 66-year-old female patient with an anterior wall acetabular fracture. **a** Anteroposterior radiograph; **b** Obturator oblique radiograph; **c** CT scan, showing femoral head injury (arrow). **d**, **e** Immediately after surgery, showing a satisfactory reduction of fracture; **f** The radiograph taken at 30 months postoperatively, showing an osteonecrosis of the femoral head (arrow)

Clinical function was excellent in 19 patients (35.8%), good in 20 (37.7%), fair in 4 (7.5%), and poor in 10 (18.9%). The average clinical score (modified Merle d’Aubigne-Postel score) was 15.6 ± 2.7 points (range: 9–18 points). Two patients in the young–old group (3.8%) underwent THA at 30 and 35 months after their initial injuries. One of the two patients have a femoral head injury and subsequent osteonecrosis of the femoral head (Fig. 1), and the other has a poor reduction and subsequent severely post-traumatic osteoarthritis.

Up to 75% of the young–old patients and 72% of the old–old patients can achieve good to excellent clinical function. The clinical scores were comparable in both groups (15.5 ± 3.2 vs. 15.7 ± 2.1, *p* = 0.787); however, comparison of the components of the modified Postel Merle D’Aubigne Score showed that the patients in the young–old group had lower pain scores (4.9 ± 1.2 vs. 5.5 ± 0.7, *p* = 0.029), higher ambulation scores (5.5 ± 1.0 vs. 4.9 ± 0.9, *p* = 0.027), and similar range of motion scores (5.1 ± 1.2 vs. 5.3 ± 0.7, *p* = 0.457) than those in the old-old group (Table 3).

**Table 3:** Clinical outcomes of both groups at the final follow-up

Variable	Young-old group (n = 28)	Old-old group (n = 25)	p-value
Clinical scores (points)	15.5 ± 3.2	15.7 ± 2.1	0.787
Degree of pain	4.9 ± 1.2	5.5 ± 0.7	0.029
Degree of ambulation	5.5 ± 1.0	4.9 ± 0.9	0.027
Range of motion	5.1 ± 1.2	5.3 ± 0.7	0.457

**Fig. 2:**
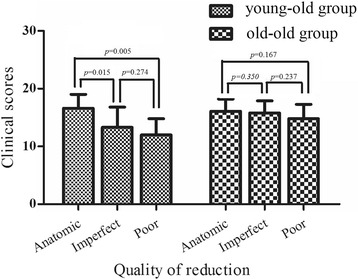
The relationship between quality of reduction and clinical scores

The average clinical scores were significantly higher in the young–old patients with anatomical reduction (16.6 ± 2.4 points; range: 9–18 points) than in those with imperfect reduction (13.3 ± 1.9 points; range: 9–18 points) (*p* = 0.015) and poor reduction (12.0 ± 2.8 points; range: 10–14 points) (*p* = 0.005, Fig. 2).

**Fig. 3:**
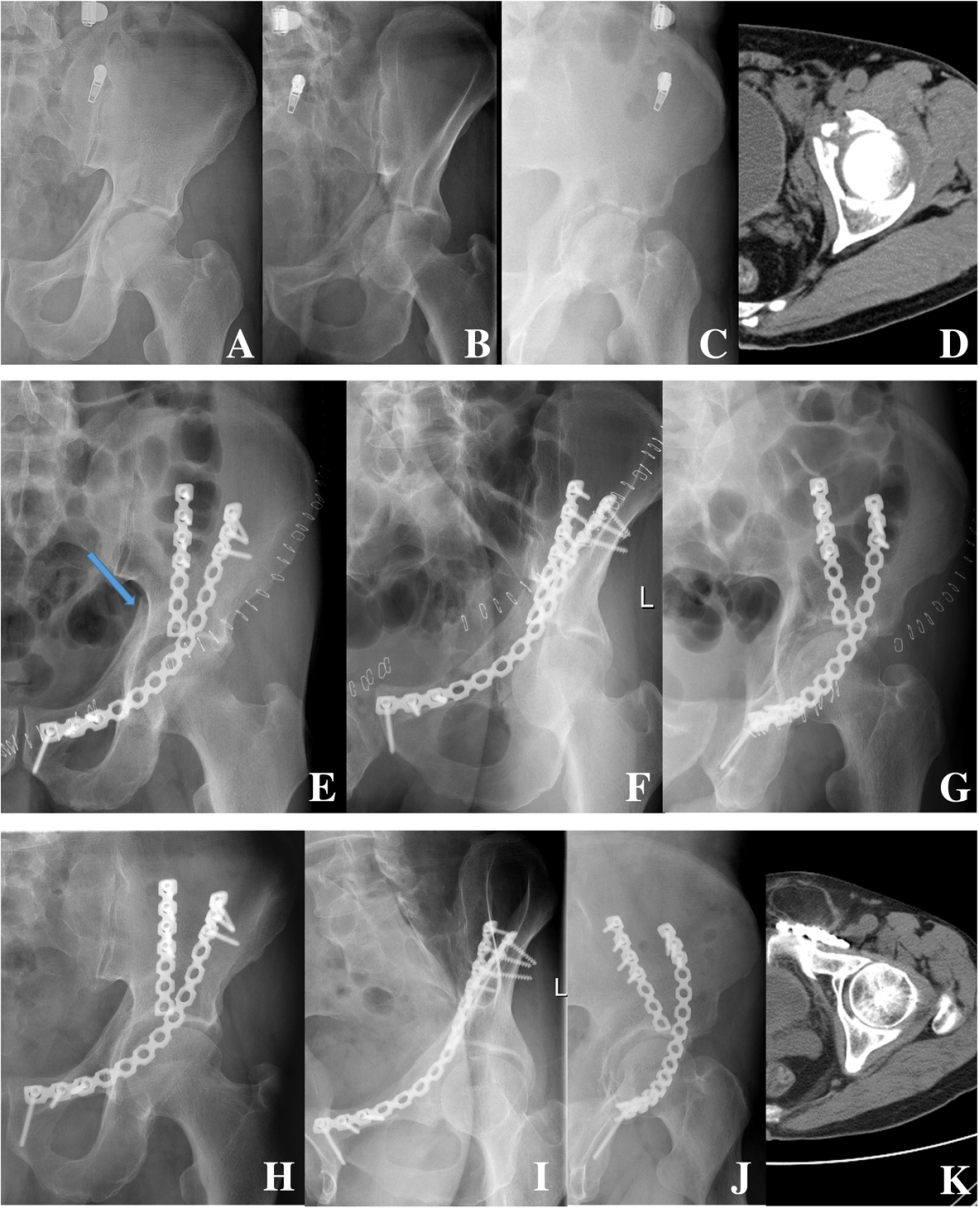
Radiographs of a 75-year-old male patient with an anterior wall acetabular fracture. **a** Anteroposterior radiograph; **b** Obturator oblique radiograph; **c** Iliac oblique radiograph; **d** CT scan. Radiographs showing imperfect reduction (arrow) of the fracture immediately after the operation. **e** Anteroposterior radiograph; **f** Obturator oblique radiograph; **g** Iliac oblique radiograph. Radiographs showing an excellent radiological outcome at 84 months after the operation and the clinical score was 18 points. **h** Anteroposterior radiograph; **i** Obturator oblique radiograph; **j** iliac oblique radiograph; **k** CT scan

The average clinical scores in the old–old patients with anatomical, imperfect, and poor reduction were 16.1 ± 2.1 (range: 12–18 points), 15.8 ± 2.1 (range: 12–18 points), and 14.8 ± 2.5 points (range: 12–18 points), respectively. No statistical difference in clinical scores was detected between the different grades in reduction quality (*p* > 0.05, Fig. 2, Fig. 3).

The rate of postoperative complication was slightly higher in the old–old group (44.0%) than in the young–old group (39.3%), but no difference was found between the groups (*p* = 0.728, Table 4). Nine (14.5%) of the 62 patients died from natural causes during the follow-up period; of these nine patients, one died at 6 months, one at 9 months, and seven at 18–72 months postoperatively (average: 35 ± 19 months). The average age at the time of surgery of the nine patients was 78.4 ± 6.3 years (range: 68–85 years).

**Table 4:** Complications and natural death during the follow-up of both groups

Variable	Young-old group (n = 28)	Old-old group (n = 25)	p-value
Complications (n, %)	11 (39.3%)	11 (44.0%)	0.728
Lateral femoral cutaneous nerve palsy	0	2 (8.0%)	
Loss of reduction	0	2 (8.0%)	
Deep venous thrombosis	1 (3.6%)	1 (4.0%)	
Superficial infection	0	1 (4.0%)	
Heterotopic ossification	8 (28.6%)	4 (16.0%)	
Grade I	3	2	
Grade II	3	2	
Grade III	2	0	
Femoral head avascular necrosis	2 (7.1%)	0	
Incisional hernia	0	1 (4.0%)	
Death (n, %)	1 (3.6%)	6 (24.0%)	0.043

### Radiological outcomes at the final follow-up

We found that better clinical function meant fewer patients willing to return for radiological evaluation. For example, only 16 patients have completed radiological evaluation. Of these patients, radiological outcomes were excellent in three patients, good in four patients, fair in three patients, and poor in six patients. The radiological outcomes do not reflect the real situation of all patients because most patients were not assessed. Thus, these outcomes were not included in this analysis.

## Discussion

With the increased acetabular fractures in the elderly population, an understanding of how these patients respond to ORIF is important. We compared the postoperative clinical function and complication rates between old–old and young–old patients who underwent ORIF and investigated the influence of patient age on the association of reduction quality and clinical function.

The clinical scores were similar in both groups (15.5 points vs. 15.7 points, *p* = 0.787). By contrast, previous studies presented an inverse relationship between age and clinical function [4–6, 9]. This discrepancy in results may be attributed to the fact that previous studies [9–11] represent a wide age variety and compare the clinical function between young patients (< 40, 50, and 55 years old) and old patients (> 40, 50, or 55 years old), whereas the present study represents only elderly patients (60–90 years) and a comparator group (young–old patients vs. old–old patients). We also compared the component of clinical score between the two groups and found higher pain scores (*p* = 0.029) and lower ambulation scores (*p* = 0.026) in the old–old patients than in the young–old patients but a comparable range of motion scores (*p* = 0.457). This result may be related to the different pain tolerance levels of patients and the increased pain threshold in the old–old patients.

Although elderly patients often have comorbid medical conditions, few age-related complications were observed and no perioperative death occurred in this study. These findings may be attributed to the rational treatment strategy for elderly patients. This strategy includes optimization of preoperative patient’s medical condition to improve surgery tolerance, as well as minimization of operative trauma and time and blood loss to achieve stable internal fixation of fractures rather than to strive for anatomical reduction. When a surgeon strives for an intraoperative anatomical reduction, this procedure often prolonged operative time and increased blood loss that may harm to the older population because older patients, especially those with medical conditions, have weaker blood loss tolerance than young patients. In the present study, the patients in the young–old group showed higher anatomical reduction (67.9% vs.36.0%, *p* = 0.024), blood loss (705 ml vs. 591 ml, *p* = 0.016), and operative time (145 min vs. 110 min, *p* = 0.021) compared with those in the old–old group. This finding may explain the similar complication rate in both groups (*p* = 0.728).

In this study, we found that reduction quality exerted an age-dependent influence on clinical function. The reduction quality in the young–old patients was significantly associated with clinical function (*p* = 0.001), and an anatomical reduction was associated with a good or excellent function. By contrast, no statistical difference was detected among the clinical functions of anatomical, imperfect, and poor reduction in the old–old patients (*p* = 0.587). We speculated that the old–old patients may have characteristics (e.g., limited life expectancy, low activity level, low functional requirements, and weak muscle strength around the hip) that may reduce the wear and tear of the hip cartilage resulting from non-anatomical reduction and may increase their tolerance to non-anatomical reduction. In addition, the follow-up time of the old–old group was shorter than that of the young–old group (43 ± 17 months vs. 61 ± 27 months) because of the limited life expectancy of the old–old patients (the death rates in the old–old and young–old groups were respectively 24.0% and 3.6%, respectively), and this study included the patients who died. In addition, studies have reported that a precise anatomical reduction highly correlates with the best long-term clinical function rather than the short-term function [9, 14, 15]. Old–old patients with the above-mentioned characteristics may not necessarily need to sacrifice operative time and blood loss to strive for anatomical reduction; a stable fracture reduction that allows early postoperative ambulation may be accepted in old–old patients. In support to our findings, Archdeacon et al. [8] conducted a retrospective study of 26 patients older than 70 years with acetabular fracture and suggested that these patients can tolerate imperfect reduction.

We found that the age-dependent influence on the rate of anatomical reduction was higher in the young–old group than in the old–old group (67.9% vs. 36.0%, *p* = 0.024) and that the loss of reduction was more likely to occur in the old–old group than in the young–old group (8.0% vs. 0%). This result may be attributed to the fact that old–old patients often have a combination of osteoporosis and comminuted acetabular fractures, which complicate the completion of anatomical reduction and stability [9]. Moreover, accurate reduction was not attempted for some of the old–old patients in this study with the aforementioned characteristics.

### Weakness of this study

This study has some limitations. First, the small sample size due to the relatively low incidence of acetabular fractures in elderly patients may compromise the robustness of the results. Second, the mean follow-up times of the young–old and old–old groups were 60.7 and 43.3 months, respectively, which may be too short to draw a definite conclusion on the association between reduction quality and clinical function, particularly in the old–old group. Third, all patients came from a single center, and the results of patients and treatment preferences from this center may not be applicable to other centers. Fourth, the clinical function was evaluated using the modified Postel Merle D’Aubigne Score instead of the patient-centered outcome scores (e.g., EQ5D, SF-12, SMFA, and SF-36). The patient-centered outcome scores for elderly patients may be much helpful. However, the modified Postel Merle D’Aubigne Score is widely applied to assess the clinical function of patients with acetabular fractures, and this scoring system has been routinely used since 1990 [16, 17].

## Conclusions

The present findings add evidence to justify performing ORIF in elderly patients with acetabular fractures. Our findings suggest that individuals older than 75 years may achieve similar clinical functions and complications to young–old patients. Moreover, unlike that in the young–old patients, the reduction quality in the old–old patients may be not affecting the clinical function. Therefore, the treatment strategy of acetabular fractures with ORIF may be different according to different age groups. In particular, the goal for young–old patients should be anatomical reduction, whereas that for old–old patients should be to minimize operative time and blood loss, and thus achieve a stable fixation that allows early post-operative ambulation.

## Additional file

Additional file 1: Detailing patients’ data. (XLSX 31 kb)

## Abbreviations

ORIF: Open reduction and internal fixation
THA: Total hip arthroplasty
